# Biotransformation of Geniposide into Genipin by Immobilized* Trichoderma reesei* and Conformational Study of Genipin

**DOI:** 10.1155/2018/2079195

**Published:** 2018-04-12

**Authors:** Yishun Yang, Yue Ding, Tong Zhang

**Affiliations:** ^1^Experiment Centre of Teaching and Learning, Shanghai University of Traditional Chinese Medicine, Shanghai 201203, China; ^2^School of Chinese Materia Medica, Shanghai University of Traditional Chinese Medicine, Shanghai 201203, China

## Abstract

*Trichoderma reesei* QM9414,* Trichoderma viride* 3.316,* Aspergillus niger* M85, and* Aspergillus niger* M92 were screened for hydrolyzing geniposide into genipin.* T. reesei* was selected according to the *β*-glucosidase activity of the fermentation broths using geniposide as a substrate.* T. reesei *was immobilized by embedding method using sodium alginate as the carrier. Geniposide was hydrolyzed by immobilized* T. reesei* at 28°C (200 rpm) for 34 h, and the yield of genipin was 89%. The product was purified and identified by UV, IR, EIMS, and ^1^H-NMR. Since there were two sets of signals in ^1^H-NMR spectra, a series of experiments were performed and verified that the existence of two conformations was the main reason. Generally, biotransformation of geniposide into genipin by immobilized* T. reesei* provides a promising solution to the genipin production.

## 1. Introduction

Genipin is a bioactive compound coming from* Gardenia jasminoides* Ellis, one kind of traditional Chinese medicine. Nowadays, various pharmacological effects including hepatoprotection [1], anti-inflammatory [2, 3], antioxidant [4], and antidepression [5] activities of this natural product have been confirmed. Due to low toxicity and excellent biocompatibility [6], it has been widely used as a natural cross-linking agent [7] for drug delivery applications [8–10] and tissue engineering [11–13]. In addition, genipin reacts spontaneously with amino and protein to form blue pigments [14], which is a promising natural colorant for the food industry [15].

**Figure 1:**
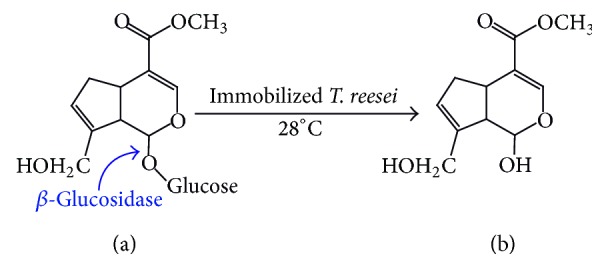
Biotransformation of geniposide (a) into genipin (b) by immobilized* T. reesei*.

As the content in Fructus Gardeniae is very low, genipin (Figure 1(b)) is prepared mainly from hydrolysis of geniposide (Figure 1(a)) by *β*-glucosidase [16]. Despite the fact that there were many papers about deglycosylation of geniposide by enzyme [17] or microorganism [18–20], to get genipin in a low cost and easy procedure is still the frontier of biochemical industry. In our previous report, geniposide was transformed into genipin by immobilized *β*-glucosidase in an aqueous-organic two-phase system [21]. Via recycling of *β*-glucosidase, the cost has been significantly reduced. However, the consumption of pure *β*-glucosidase, which is expensive and hard to handle, is a barrier in the way of bulky production.

Fungi have long been considered to be the most productive strains for *β*-glucosidase production [22–25]. In this paper, four fungi strains (*Trichoderma reesei* QM9414,* Trichoderma viride* 3.316,* Aspergillus niger* M85, and* Aspergillus niger* M92) were investigated for their ability to produce *β*-glucosidase. A simple method using geniposide as a substrate was applied to measure the activity of *β*-glucosidase [26].* T. reesei* showed the highest *β*-glucosidase activity against geniposide. It was immobilized by embedding method and applied to transform geniposide into genipin.

The structure of the genipin product was identified by UV, IR, EIMS, and ^1^H-NMR. However, two compounds were observed in ^1^H-NMR spectra, which could not be eliminated by purification like recrystallization and drying. We hypothesized that the existence of conformational isomer or configurational isomer of genipin results in this issue. Configurational isomers of the same pharmaceutical have the same physicochemical properties, but may differ in pharmacokinetics, physiological activities, and toxicity [27]. A series of experiments were performed to verify whether configurational isomer or conformational isomer caused genipin's second set of signals in ^1^H-NMR spectra.

## 2. Materials and Methods

### 2.1. Materials and Reagents

Geniposide was extracted from gardeniae fruits (the purity was about 88% by HPLC). Standard geniposide and standard genipin were purchased from National Institutes for Food and Drug Control (Beijing, China).* p*-Nitrophenyl-*β*-D-glucoside (*p*NPG) was purchased from Duly Biotechnology Co. Ltd. (Nanjing, China). Sodium alginate and other reagents were purchased from Sinopharm (Beijing, China).

### 2.2. Microorganism and Culture Media

*T. reesei *QM9414 (ATCC 26921),* T*.* viride *3.316,* A. niger *M85, and* A. niger *M92 were purchased from Shanghai Industrial Microbiology Institute (Shanghai, China).

1 L Mandels medium (MM medium) contained 20 g glucose, 1 g tryptone, 0.3 g (NH_4_)_2_SO_4_, 2 g KH_2_PO_4_, 0.4 g CaCl_2_·2H_2_O, 0.02 g MgSO_4_·7H_2_O, 5 g FeSO_4_·7H_2_O, 1.6 mg MnSO_4_·H_2_O, 1.4 mg ZnSO_4_·7H_2_O, 3.7 mg CoCl_2_·6H_2_O, and 50 mL citrate buffer (1 M, pH 4.5), and the pH was adjusted to 4.8 [28]. 1 L MM fermentation medium (MMF medium) contained the same composition as MM medium except that carbon source (glucose) was replaced with 10 g sodium carboxymethyl cellulose. 1 L immobilized cell medium (IC medium) contained 20 g wheat bran, 1 g tryptone, 0.5 g CaCl_2_, and 1 g KH_2_PO_4_. 1 L geniposide fermentation medium (GF medium) was similar to IC medium except that 1 g geniposide was added.

### 2.3. Determination of *β*-Glucosidase Activity

#### 2.3.1. *p*NPG Method

The activity of *β*-glucosidase (per unit) was defined as moles of* p*NPG converted by 1 mL enzyme solution per minute [29]. The determination steps were as follows [21]: 10 *μ*L *β*-glucosidase solution, 50 *μ*L* p*NPG solution (4 mM), and 40 *μ*L sodium acetate buffer (0.2 M, pH 4.5) were mixed and incubated in water bath (50°C) for 10 min. Then the reaction was stopped by adding 100 *μ*L Na_2_CO_3_ solution (1 M). The reaction was allowed to cool at room temperature and the absorbance at 400 nm was measured using a microplate reader (Eon, BioTek Instruments, Inc., USA).

#### 2.3.2. GPS Method

Genipin cross-links with amine, amino acid, and protein to form a kind of blue pigments (gardenia blue) [30], which can be used to determine the content of genipin. GPS method was used to measure *β*-glucosidase activity as described by Liang et al. [26]. The activity of *β*-glucosidase (per unit) was defined as moles of geniposide converted by 1 mL enzyme solution per minute. The determination steps were as follows: 10 *μ*L *β*-glucosidase solution, 50 *μ*L geniposide solution (4 mM), and 40 *μ*L sodium acetate buffer (0.2 M, pH 4.5) were mixed and incubated in water bath (50°C) for 30 min. After that, 80 *μ*L glycine (0.2 mg/mL) was added to the mixture and incubated in boiling water bath for 10 min. The reaction was allowed to cool at room temperature and the absorbance at 590 nm was measured using a microplate reader.

### 2.4. Screening for *β*-Glucosidase Production Strains

*T. reesei*,* T*.* viride*,* A. niger *M85, and* A. niger *M92 were inoculated on potato dextrose agar (PDA) plates at 28°C for 3−5 days. Spores were suspended with saline. Erlenmeyer flasks (250 mL) containing 50 mL MMF medium were inoculated with* T. reesei*,* T*.* viride*,* A. niger *M85, and* A. niger *M92 spore suspension at a final concentration of 1 × 10^8^ spores/L, respectively. The culture was incubated in a shaking incubator at 28°C (200 rpm). After being incubated for 5 days, the fermentation medium was centrifuged at 10000 rpm for 10 min at 4°C. The supernatants were collected as the enzyme solutions and the *β*-glucosidase activity was measured by GPS method.

### 2.5. Immobilization of* T. reesei*

*T. reesei* cells were immobilized by the embedding method using sodium alginate as the carrier.* T. reesei* were inoculated on PDA plates at 28°C for 3−5 days. Spores were suspended with saline. Erlenmeyer flasks (250 mL) containing 100 mL MM medium were inoculated with* T. reesei* spore suspension at a final concentration of 1 × 10^8^ spores/L. The culture was incubated in a shaking incubator at 28°C (200 rpm). After being incubated for 5 days, the fermentation medium was centrifuged at 4000 rpm for 10 min. The* T. reesei* cells were collected and dried with sterile filter paper and then weighed and suspended with sterile water.* T. reesei* cell suspension was mixed with sterile sodium alginate solution. The mixture was added dropwise to CaCl_2_ solution at the height of 10 cm and stood for 0.5−2.5 h at 4°C. The gel beads were washed with sterile water and stored at 4°C.

### 2.6. Cultivation of Immobilized* T. reesei*

10 g immobilized* T. reesei* was added to erlenmeyer flasks (250 mL) containing 50 mL IC medium and incubated in a shaking incubator at 28°C (200 rpm). After being incubated for 72 h, *β*-glucosidase activity of the fermentation broth was measured by GPS method.

### 2.7. Hydrolysis of Geniposide by Immobilized* T. reesei*

10 g immobilized* T. reesei* was added to erlenmeyer flasks (250 mL) containing 50 mL GF medium and incubated in a shaking incubator at 28°C (200 rpm) for 48 h. 0.5 mL fermentation broth was taken out every 2 h and stored at 4°C before use. Meanwhile, free* T. reesei* was incubated in IC medium and GF medium under the same condition. 0.5 mL fermentation broth was taken out at predetermined time intervals. After being incubated for 48 h, the fermentation broth was centrifuged at 10000 rpm for 10 min. The concentration of geniposide and genipin in the supernatants was determined using HPLC.

(1) Yield  of  genipin%=Produced  genipin  amountmolInitial  geniposide  amountmol×100%.

### 2.8. Determination of Geniposide and Genipin

The content of geniposide and genipin was determined by Agilent 1200 HPLC Systems (Agilent Technologies, USA) equipped with Diamonsil C_18_ chromatographic column (5 *μ*m, 4.6 mm × 250 mm, Dikma, China) [16]. The fermentation broths were diluted with methanol and filtered by 0.45 *μ*m microporous filtering membrane before injection. The assay was carried out after injecting 20 *μ*L sample to HPLC with column temperature of 30°C and the detection wavelength of 238 nm. The mobile phase consists of acetonitrile and water with a ratio of 15 : 85 (v/v) and a flow rate of 1 mL/min.

### 2.9. Preparation of Genipin

10 g immobilized* T. reesei* was added to erlenmeyer flasks (250 mL) containing 50 mL GF medium and incubated in a shaking incubator at 28°C (200 rpm) for 34 h. The fermentation broth was centrifuged at 10000 rpm for 10 min. Afterwards, the supernatant was extracted by ethyl acetate 3 times (50 mL each time). The organic phase (ethyl acetate) was collected and the solvent was evaporated under reduced pressure. The extract was chromatographed on silica gel with a petroleum ether, ethyl acetate solvent system (80 : 20, 75 : 25, 66 : 33), to yield genipin. The product was recrystallized by ethyl acetate. Crystals were collected and dried in vacuum drying oven for 5 h.

### 2.10. Identification of Genipin

The structure of the product (genipin) was analyzed by infrared spectrum (ATR-FTIR, Nicolet iS 10, Thermo Fisher Scientific Inc., USA), UV-visible spectrophotometer (8454, Agilent Technologies, USA), ^1^H-NMR in CDCl_3_ (AVANCE 400 MHz, Bruker, Germany), and LC/MS (Agilent 6460 Series Triple Quadrupole Systems, Agilent Technologies, USA).

### 2.11. Conformational Study of Genipin

#### 2.11.1. ^1^H-NMR Analysis of Genipin and Geniposide

Genipin was analyzed by ^1^H-NMR in CDCl_3_, DMCO-d_6_, C_5_D_5_N, and DMSO-d_6_, respectively. In addition, variable temperature ^1^H-NMR experiment of genipin was performed at 0, 25, and 50°C in CDCl_3_. Geniposide was analyzed by ^1^H-NMR in CDCl_3_.

#### 2.11.2. Acetylation of Genipin

80 mg genipin, 2 mL pyridine, and 4 mL acetic anhydride were added to a 10 mL reaction flask. The mixture was then allowed to stir at room temperature for 12 h and then neutralized with 1 M HCl. The organic phase was separated, and the aqueous layer was extracted with ethyl acetate and concentrated. The crude product was purified by Sephadex LH-20 (eluted by methanol) and column chromatography on silica gel (eluted by petroleum ether : acetone = 20 : 1) to afford acetylation of genipin. Acetylated genipin was analyzed by ^1^H-NMR in C_5_D_5_N.

#### 2.11.3. Separation of Genipin's Configurational Isomer by HPLC Equipped with Chiral Column

Genipin's configurational isomer was determined by Waters 515 HPLC system (DAD detector, Waters Corporation, USA) using CHIRALCEL OD-H and AD-H chiral columns (5 *μ*m, 4.6 mm × 250 mm, Daicel Chiral Technologies, Japan). The flow rate of the mobile phase was 0.6 mL/min, and the composition of the mobile phase is shown in Table 1.

### 2.12. Single-Crystal X-Ray Diffraction of Genipin

Slow evaporation from an acetone solution at room temperature led to the formation of single crystals suitable for analysis by single-crystal X-ray diffraction method. The single-crystal X-ray diffraction was recorded at 293(2) K on a Bruker SMART CCD diffractometer using molybdenum K*α*1 radiation (*λ* = 0.71073 Å). The structure was solved by direct methods with a SHELXTL-97 software package [31]. The crystallographic data of genipin are shown in Table S1. CCDC 1574854 contains supplementary crystallographic data for this paper. These data can be obtained free of charge from the Cambridge Crystallographic Date Center via https://www.ccdc.cam.ac.uk.

### 2.13. Statistical Analysis

All experiments were performed in triplicate, and the data are presented as the mean ± standard deviation (SD). Differences between groups were analyzed by ANOVA. Differences were considered significant when *p* < 0.05.

## 3. Results and Discussion

### 3.1. Screening for *β*-Glucosidase Production Strains

With high sensitivity and easy procedure,* p*NPG method was widely used to determine the activity of *β*-glucosidase [32]. However, because of substrate specificity, there are significant differences of *β*-glucosidase activity against different substrates [33, 34]. Genipin cross-links with amine, amino acid, and free amine group of protein and generates blue pigments [30, 35], which can be used to determine the content of genipin. Though geniposide was added in the culture plate to screen microorganisms for geniposide hydrolysis as reported by Xu et al. [36], the result was in a qualitative manner. Liang et al. [26] reported that genipin reacted with glycine to form blue pigments with maximum absorption at around 590 nm, and thereby developed a method to measure *β*-glucosidase activity. In this paper, this method was applied to screen the strains of high geniposide transformation activity quantitatively.

**Figure 2:**
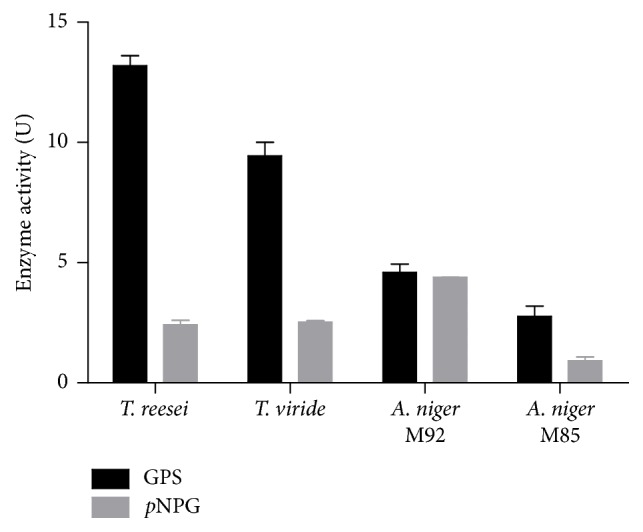
*β*-Glucosidase activity of the fermentation broths.

Four fungi strains were tested for their ability to produce *β*-glucosidase. As shown in Figure 2, *β*-glucosidase activities of the fermentation broths against geniposide and* p*NPG were determined by GPS method and* p*NPG method as described in Section 2.3, respectively. All strains revealed the ability to produce *β*-glucosidase.* T. reesei *has demonstrated a competitive capability among the four strains when using geniposide as a substrate. Interestingly, the enzyme activities on* p*NPG and geniposide were significantly different due to substrate specificity. The *β*-glucosidase activity of* T. reesei* fermentation medium against geniposide was 5.5 times as high as against* p*NPG. However, when using* p*NPG as a substrate, the *β*-glucosidase activity of* A. niger *M92 fermentation medium was the highest (Figure 2). Since the objective of the screening is to find a *β*-glucosidase which is able to hydrolyze geniposide efficiently,* T. reesei* was chosen for the subsequent experiments.

### 3.2. Optimization of Immobilization Conditions

Immobilization conditions, including sodium alginate concentration, CaCl_2_ concentration, immobilization time, and cell concentration, were investigated. Immobilized* T. reesei* were cultivated in IC medium at 28°C (200 rpm) for 72 h. Then *β*-glucosidase activity of the fermentation broths was measured by GPS method.

**Figure 3:**
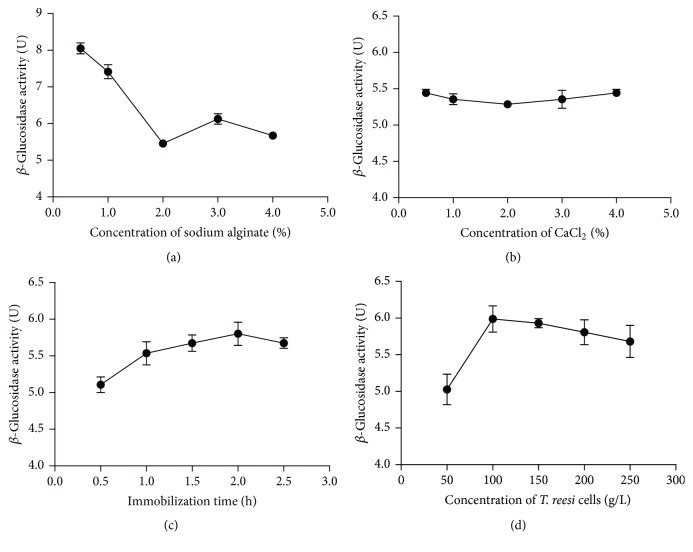
Optimization of immobilization conditions. (a) Influence of sodium alginate concentration, (b) CaCl_2_ concentration, (c) immobilization time, and (d) cell concentration on *β*-glucosidase activity of immobilized* T. reesei*.

Sodium alginate solutions of different concentrations (0.5−4.0%, w/v) were used to embed* T. reesei* cells. As shown in Figure 3(a), the enzyme activity was the highest at a sodium alginate concentration of 0.5%. However, the gel beads were friable when the sodium alginate concentration ranged from 0.5 to 2.0%, and the beads crushed during fermentation. When the sodium alginate concentration ranged from 2.0 to 4.0%, the enzyme activity reached another peak at 3%. And hardness of the gel beads was good at a sodium alginate concentration of 3%.

CaCl_2_ solutions of different concentration (0.5−4.0%, w/v) were used to replace the Na^+^ in the surface of the gel beads. The results are shown in Figure 3(b); CaCl_2_ concentration ranging from 0.5 to 4.0% had little effect on *β*-glucosidase activity. But the replacement rate of the Na^+^ in the surface of gel beads increased with the CaCl_2_ concentration, and the Na^+^ in the surface were replaced rapidly before the replacement of internal Na^+^ at a high CaCl_2_ concentration, which led to incomplete embedding and low hardness inside the gel beads. Thus, when the CaCl_2_ concentration exceeded 2.0%, the gel beads became fragile and the cells were embedded incompletely.

The immobilized* T. reesei* gel beads were immobilized in CaCl_2_ solution for 0.5−2.5 h. As shown in Figure 3(c), the enzyme activity increased with immobilization time at 0.5−2.0 h and peaked at 2.0 h.* T. reesei* cells suspension of different concentrations (50−250 g/L) was prepared and immobilized. The result (Figure 3(d)) showed that the enzyme activity increased with cell concentration at 50−100 g/L. But the enzyme activity decreased with cell concentration at 100−250 g/L. It was probably because the growth of the cell was restricted if the cell density in the gel beads was too high.

In summary, the optimized immobilization procedure was as follows:* T. reesei* cells suspension (100 g/L) was mixed with sterile sodium alginate solution (3%). The mixture was added dropwise to CaCl_2_ solution (0.5%) at the height of 10 cm and stood for 2 h at 4°C. The gel beads were washed with sterile water and stored at 4°C.

### 3.3. Reusability of Immobilized* T. reesei*

**Figure 4:**
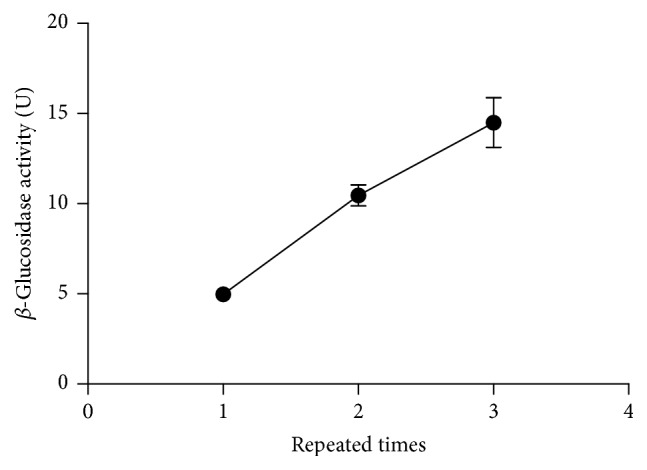
Influence of used times on *β*-glucosidase activity of immobilized* T. reesei*.

Immobilized* T. reesei *were cultivated in IC medium at 28°C (200 rpm) for 72 h. Then the immobilized* T. reesei* gel beads were separated and reused under the same condition 3 times. *β*-Glucosidase activity of the fermentation broth was measured by GPS method each time. The enzyme activity usually decreases with recycle times [37]. On the contrary, the result (Figure 4) showed that the enzyme activity increased significantly with repeated times (*p* < 0.01). Genipin is known as an effective cross-linker [7]. The produced *β*-glucosidase might cross-link with the carrier (calcium alginate gel) by genipin, which decreased the deactivation and leakage of *β*-glucosidase during the repeated reactions. Consequently, the activity of immobilized* T. reesei* increased (2.9-fold as high as the first time after being reused 3 times) because of the accumulation of *β*-glucosidase. Fujikawa et al. [17] reported similar immobilization of *β*-glucosidase in calcium alginate gel using genipin as a cross-linker. Furthermore, the result indicated that the viability of* T. reesei* was good in the immobilized environment.

### 3.4. Biotransformation of Geniposide by Immobilized* T. reesei*

**Figure 5:**
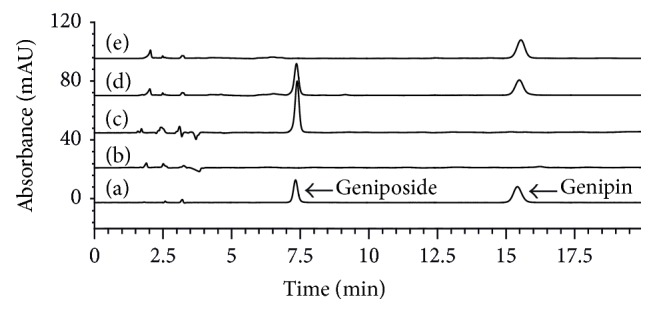
HPLC chromatograms of (a) geniposide and genipin standard, (b) IC medium after being cultivated by immobilized* T. reesei* for 48 h, (c) initial GF medium, (d) GF medium after being cultivated by immobilized* T. reesei* for 24 h, and (e) GF medium after being cultivated by immobilized* T. reesei* for 48 h.

Immobilized* T. reesei* were cultivated in GF medium containing 1 g/L geniposide at 28°C (200 rpm) for 48 h. 0.5 mL fermentation broth was taken out every 2 h from 22 h to 48 h and measured by HPLC. HPLC chromatograms are shown in Figure 5.

**Figure 6:**
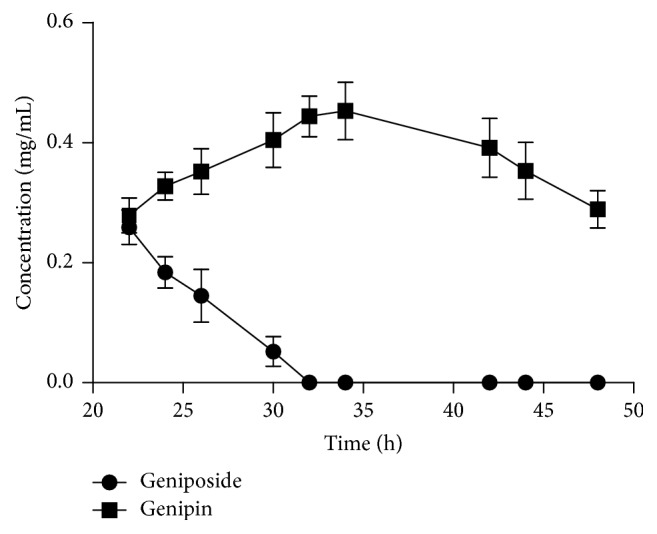
Concentration curves of geniposide and genipin during biotransformation of geniposide by immobilized* T. reesei*.

As shown in Figure 6, the amount of geniposide declined in the first 30 h, and geniposide was undetected after 32 h. In contrast, the amount of genipin exhibited an opposite trend within 34 h. The result demonstrated that the geniposide was completely transformed into genipin within 34 h. The amount of genipin reached the peak at 34 h, with the yield of 89%. Nevertheless, the yield of genipin fell from 89% at 34 h to only 56% at 48 h. The possible reason was that genipin reacted with amino and protein in the medium and formed a lot of byproducts after 34 h. Geniposide was hydrolyzed with free* T. reesei* under the same condition for 0−120 h and the yield of genipin was 24%, 60%, and 73% at 72 h, 96 h, and 120 h, respectively. The data revealed that immobilization of* T. reesei* can dramatically enhance the production efficiency of genipin.

### 3.5. Identification of Genipin

The purity of produced genipin was over 98% by HPLC. The analytical results of the product are as follows: UV (MeOH) *λ*_max_ 239 nm (Figure S1) [38]; IR (cm^−1^): 3387.79, 3202.75, 1677.85, 1617.74, 1440.43, and 1297.90 (Figure S2) [39]; EIMS* m/z* 225 [M]^−^ (Figure S3); ^1^H-NMR (Figure S4) data shown in Table 2 [36]. The data above are in accordance with previous reports.

### 3.6. Conformational Isomer Study of Genipin

**Figure 7:**
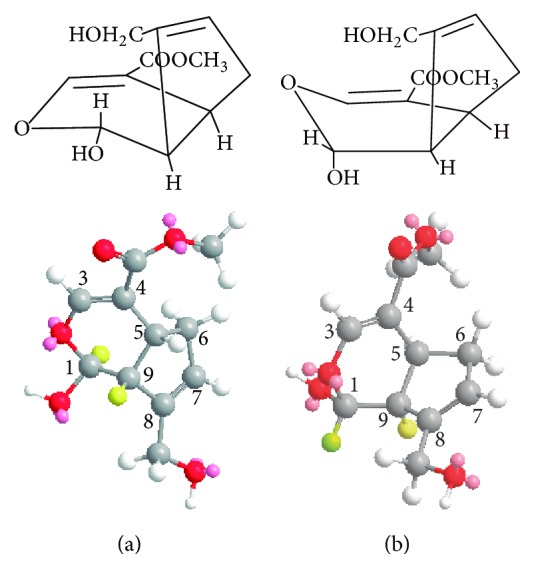
Conformations of genipin in position 1 (adapted with permission from [40]). (a) GI: ∠H_1_H_9_ ≈ 180°, *J*_1,9_ = 7–10 Hz; (b) GII: ∠H_1_H_9_ ≈ 90°, *J*_1,9_ = 0−2 Hz.

**Figure 8:**
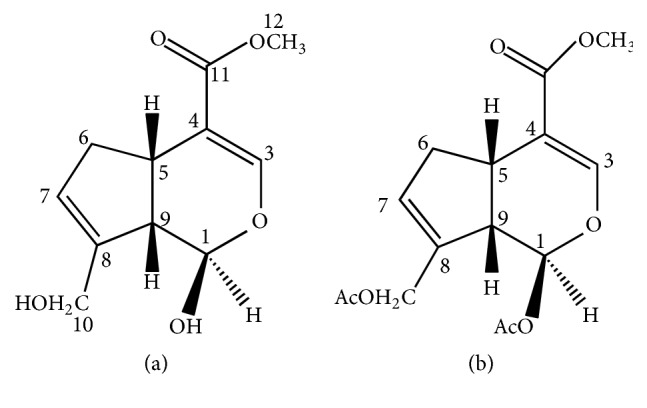
Chemical structure of (a) genipin and (b) acetylated genipin.

The produced genipin was analyzed by ^1^H-NMR, and two compounds were observed in ^1^H-NMR spectra (Figure S4). The spectrum of the major signals (GI) is identified as genipin according to a previous report [40]. But as far as we know, the spectrum of the minor signals (GII) is unreported. Genipin obtained from commercial source (purity > 98%, Linchuan Zhixin Biotechnology Co. Ltd., China) was analyzed by ^1^H-NMR, and the spectrum was similar. Several purification methods such as recrystallization and drying were performed but GII still existed. It was reported that some iridoid compounds may have existed as two conformations known as ^9^T_1_ conformation (Figure 7(a)) and ^1^T_9_ conformation (Figure 7(b)) in dihydropyran ring [41]. Although Djerassi et al. [40] have mentioned that genipin has two conformations (Figure 7), unfortunately, the structures of them have not been confirmed. Hence, it is reasonable to doubt that the existence of two conformations of genipin caused the appearance of two products. However, there is a chiral carbon atom in position 1 (Figure 8(a)), in which the glycosyl is replaced by hydroxy during the process of geniposide deglycosylation. Therefore, it is possible to form configurational isomer of genipin in the production process.

To exclude the existence of configurational isomer in position 1, the produced genipin was analyzed by HPLC using chiral column to separate its configurational isomer, but no additional peak was observed (Figure S5). Moreover, acetylated genipin (Figure 8(b)) was determined by ^1^H-NMR, and single set of signals appeared (Figure S6). If genipin's configurational isomer existed, there would be two corresponding acetylated products. When the hydroxy in position 1 is acetylated, the substituent inclined in key e as acetyl is larger than hydroxy and it becomes instable in key a. Furthermore, the energy barrier between key e and key a rises, which is adverse to the interconversion of conformations. As a consequence, acetylated genipin only exists as GI. The ^1^H-NMR analysis of geniposide showed that there was only one set of signals (Figure S7, *J*_1–9_ = 7.8 Hz). There is a glucosyl linking to carbon atom 1 in geniposide molecule. As the glucosyl is even larger than acetyl, it is much more stable in key e. This result agrees with our assumption. These results indicated that there was no configurational isomer of genipin formed.

^1^H-NMR data of genipin are shown in Table 2. The dihedral angle between H-1 and H-9 of these two conformations was different; therefore the coupling constants of H-1 and H-9 can be used to determine the dominant conformation. We observed that *J*_1–9_ = 8.4 Hz in the major signals (GI), while *J*_1–9_ = 2.8 Hz in the minor signals (GII). Therefore, we deduced that GI is the dominant conformation of genipin. As the hydroxy of GI in position 1 is in key e while the hydroxy of GII is in key a, the energy of GII is higher than GI, so that GI is more stable than GII.

**Figure 9:**
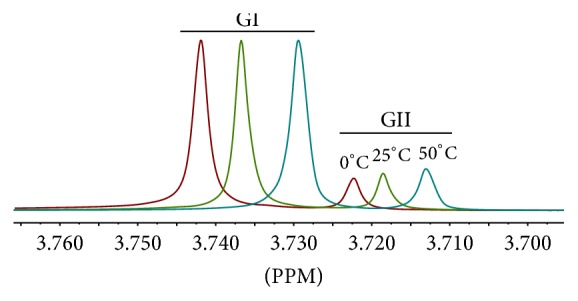
^1^H-NMR spectra of genipin at 0, 25, and 50°C in CDCl_3_.

^1^H-NMR spectra of genipin in different organic solvents and at different temperatures were investigated. Tommaso et al. [42] reported the formation of genipin configurational isomers induced by ring-opening reaction in aqueous solution at different pHs. Configurational isomers of genipin may form by the pH change in the production or NMR measurement. However, the interconversion of genipin configurational isomers is catalyzed by water. As the ^1^H-NMR experiments were performed in anhydrous solvents in this study, the mutarotation reaction of genipin was unlikely to happen without water as a catalyst. Provided that the other product is configurational isomer of genipin formed by the pH change in the production, the integral proportions of these two sets of signals would be constant in different organic solvents, and the ratio of these isomers should not be affected by temperatures. The results showed that the integral proportion of these two isomers varied with solvents (Table 3) and temperatures (Table 4). These spectra are shown in Figures S4 and S8−S10. It is known that the quantitative proportions of conformational isomers are influenced by solvents and temperatures. Moreover, the integral ratio of GI decreased as the temperature rose. It is probably because the energy of GII is higher than GI, so the content of GII increased with the temperature (Figure 9). The above results verified that these two products were conformational isomers.

**Figure 10:**
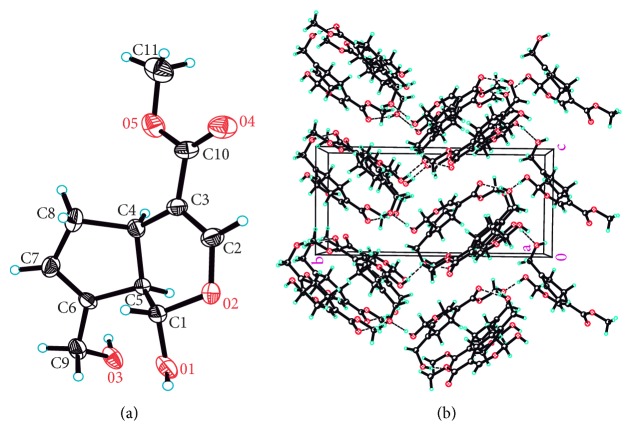
Crystal structure of genipin. (a) ORTEP view; (b) molecular packing diagram.

To further confirm the preferred conformation, molecular structure of genipin was obtained by single-crystal X-ray diffraction analysis. The molecular structure of genipin is in accordance with the theoretical preferred conformation (GI) based on our hypothesis and more stable crystal form reported previously [43]. Crystal structure of genipin is shown in Figure 10. Crystal data and structure refinement of genipin are shown in Table S1.

## 4. Conclusions

Four kinds of commercial fungi strains (*T. reesei*,* T*.* viride*,* A. niger *M85, and* A. niger *M92) were screened for enzymatic deglycosylation of geniposide by GPS method using geniposide as a substrate.* T. reesei* was selected and immobilized by embedding method using sodium alginate as the carrier. The immobilization procedure was easily handled and the reusability was excellent. Geniposide was hydrolyzed by immobilized* T. reesei* at 28°C (200 rpm) for 34 h, with the yield up to 89%, compared to 73% at 120 h by free* T. reesei*. The genipin product was purified by silica gel chromatography and recrystallization. The structure of the genipin (purity > 98%) was identified by UV, IR, EIMS, and ^1^H-NMR. As two products occurred in ^1^H-NMR spectrum of genipin, it was analyzed by ^1^H-NMR and separated by HPLC equipped with chiral column. The analysis results indicated that conformational isomer rather than configurational isomer results in the appearance of the second set of ^1^H-NMR signals. Through single-crystal X-ray diffraction analysis, GI was confirmed as a preferred conformation. In conclusion, the biotransformation of geniposide into genipin by immobilized* T. reesei* is an economical and efficient method to produce genipin.

## Figures and Tables

**Table 1:** HPLC conditions of separation of genipin's configurational isomer using chiral columns.

Entry	Column	Mobile phase
1	OD-H	Cyclohexane–isopropanol−diethylamine (80 : 20 : 0.025)
2	OD-H	Cyclohexane–isopropanol–diethylamine (65 : 35 : 0.5)
3	AD-H	Cyclohexane–isopropanol–diethylamine (65 : 35 : 0.5)

**Table 2:** ^ 1^H-NMR spectroscopic data of genipin in CDCl_3_.

Number	H's	GI	GII
		*δ*_H_ (*J* in Hz)	*δ*_H_ (*J* in Hz)
1	1	4.83, d (*J*_1–9_ = 8.4)	5.28, d (*J*_1–9_ = 2.8)
3	1	7.54, s	7.50, s
5	1	3.22, m	3.22, m
6a	1	2.90, ddt (*J*_6a-6b_ = 16.7, *J*_6a–7_ = 8.6, *J*_6a-5_ = 1.9)	2.74, dd (*J*_6a-6b_ = 17.3, *J*_6a–7_ = 7.9)
6b	1	2.08, ddt (*J*_6b-6a_ = 16.7, *J*_6b-7_ = 9.4, *J*_6b–5_ = 2.0)	2.30, d (*J*_6b-6a_ = 17.3)
7	1	5.90, s	5.83, s
9	1	2.55, m	3.37, td (*J*_9–5_ = 8.1, *J*_9–1_ = 2.8)
10	2	4.33, m	4.33, m
12	3	3.74, s	3.72, s

**Table 3:** Proportion of genipin isomers in different solvents in ^1^H-NMR spectra.

Entry	Solvent	Integral ratio (GI : GII)
1	CDCl_3_	4.51 : 1
2	DMCO-d_6_	7.11 : 1
3	C_5_D_5_N	7.09 : 1
4	DMSO-d_6_	9.85 : 1

**Table 4:** Proportion of genipin isomers at different temperatures in ^1^H-NMR spectra.

Entry	Temperature (°C)	Integral ratio (GI : GII)
1	0	5.11 : 1
2	25	4.60 : 1
3	50	3.64 : 1

The solvent was CDCl_3_.

## Supplementary Materials

Supplementary Materials Figure S1: UV-vis absorption spectrum of genipin, and the solvent was MeOH. Figure S2: FTIR spectrum of genipin. Figure S3: EIMS spectrum of genipin. Figure S4: ^1^H-NMR spectrum of genipin, and the solvent was CDCl_3_. Figure S5: HPLC chromatograms of genipin using chiral columns under different conditions. Figure S6: ^1^H-NMR spectrum of acetylated genipin, and the solvent was C_5_D_5_N. Figure S7: ^1^H-NMR spectrum of geniposide, and the solvent was CDCl_3_. Figure S8: ^1^H-NMR spectrum of genipin, and the solvent was DMCO-d_6_. Figure S9: ^1^H-NMR spectrum of genipin, and the solvent was C_5_D_5_N. Figure S10: ^1^H-NMR spectrum of genipin, and the solvent was DMSO-d_6_. Table S1: the crystallographic data of genipin.
