# Supplementary material for: Biotransformation of Geniposide into Genipin by Immobilized Trichoderma reesei and Conformational Study of Genipin
Source: Biomed Res Int. 2018 Apr 12;2018:2079195. doi: 10.1155/2018/2079195 (PMC5925029; doi:10.1155/2018/2079195)
Supplement: Supplementary Materials — Figure S1: UV-vis absorption spectrum of genipin, and the solvent was MeOH. Figure S2: FTIR spectrum of genipin. Figure S3: EIMS spectrum of genipin. Figure S4: 1H-NMR spectrum of genipin, and the solvent was CDCl3. Figure S5: HPLC chromatograms of genipin using chiral columns under different conditions. Figure S6: 1H-NMR spectrum of acetylated genipin, and the solvent was C5D5N. Figure S7: 1H-NMR spectrum of geniposide, and the solvent was CDCl3. Figure S8: 1H-NMR spectrum of genipin, and the solvent was DMCO-d6. Figure S9: 1H-NMR spectrum of genipin, and the solvent was C5D5N. Figure S10: 1H-NMR spectrum of genipin, and the solvent was DMSO-d6. Table S1: the crystallographic data of genipin. [file 2079195.f1.pdf]

Supporting Information

Biotransformation of geniposide into genipin by immobilized *Trichoderma reesei* and conformational study of genipin

Yishun Yang [1,2], Yue Ding [1], Tong Zhang [1]

[1]*Experiment Centre of Teaching and Learning, Shanghai University of Traditional Chinese Medicine, Shanghai 201203, China*

[2]*School of Chinese Materia Medica, Shanghai University of Traditional Chinese Medicine, Shanghai 201203, China*

Correspondence should be addressed to Tong Zhang; zhangtdmj@hotmail.com.

Figure S1: UV-VIS spectrum of genipin in MeOH.

Figure S2: FTIR spectrum of genipin.

Figure S3: EIMS spectrum of genipin.

Figure S4**:** ^{1}H-NMR spectrum of genipin in $CDCl_3$.

Figure S5: HPLC chromatograms of genipin using chiral columns under different conditions.

Figure S6: ^{1}H-NMR spectrum of acetylated genipin in C_5D_5N.

Figure S7: ^{1}H-NMR spectrum of geniposide in $CDCl_3$.

Figure S8: ^{1}H-NMR spectrum of genipin in DMCO-d_6.

Figure S9: ^{1}H-NMR spectrum of genipin in C_5D_5N.

Figure S10: ^{1}H-NMR spectrum of genipin in DMSO-d_6.

Table S1: Crystallographic data of genipin.

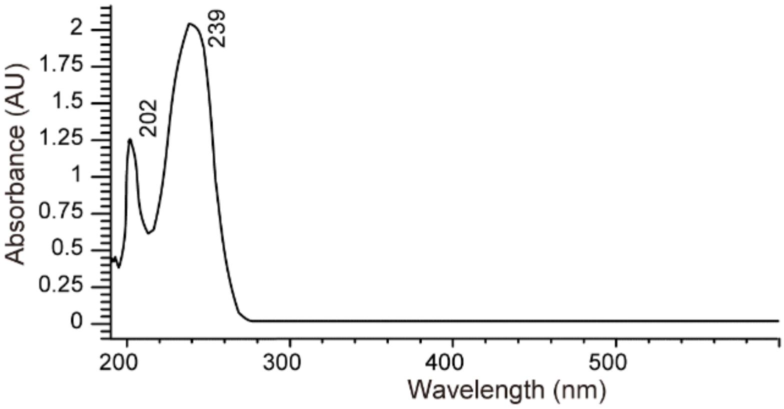

FIGURE S1: UV-VIS spectrum of genipin in MeOH.

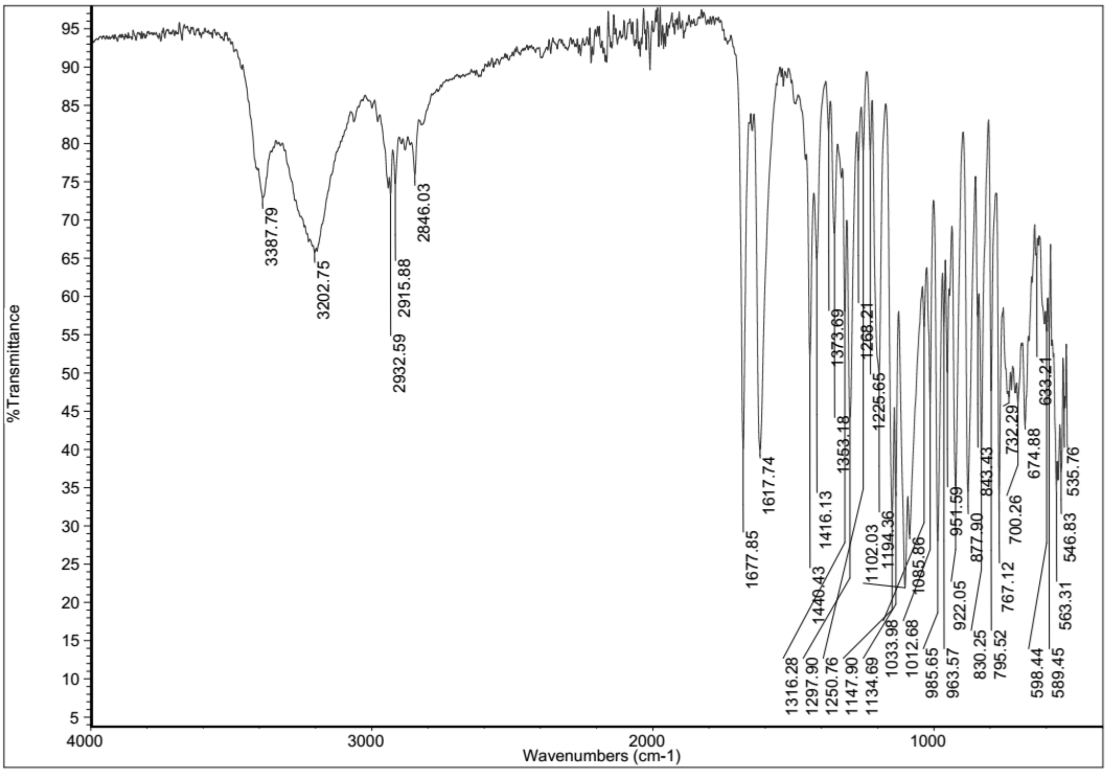

FIGURE S2: FTIR spectrum of genipin.

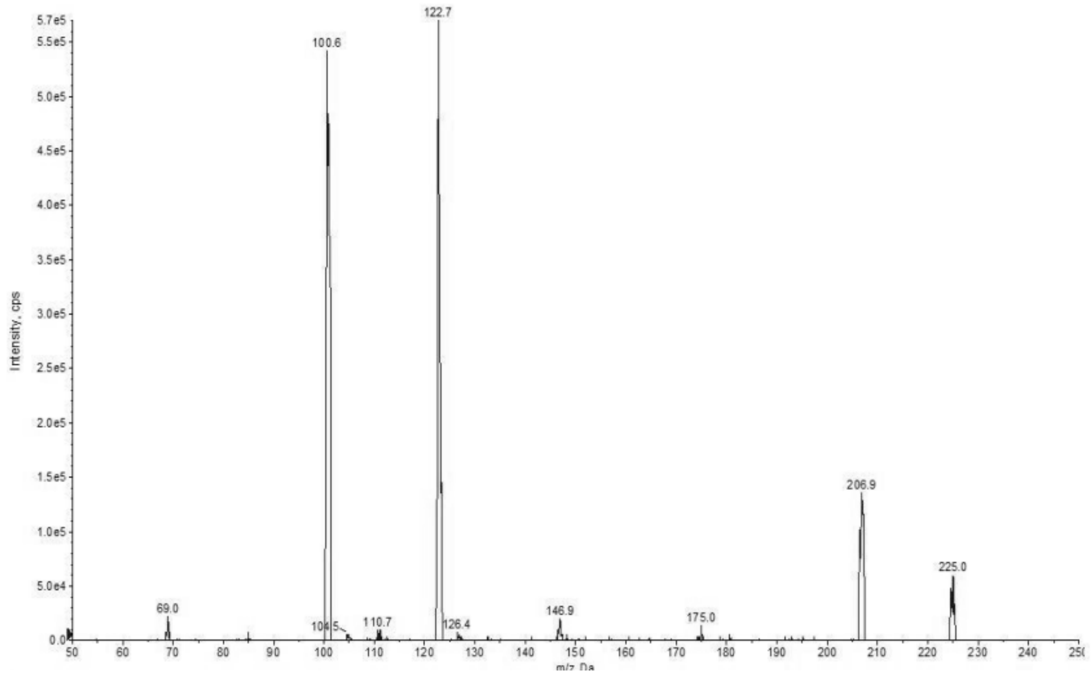

FIGURE S3: EIMS spectrum of genipin.

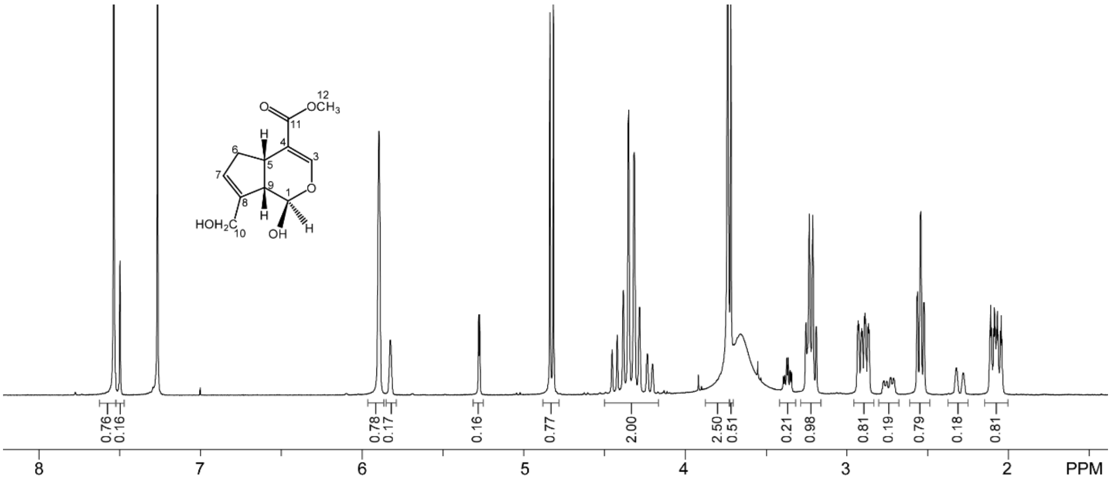

FIGURE S4: ^{1}H-NMR spectrum of genipin in $CDCl_3$.

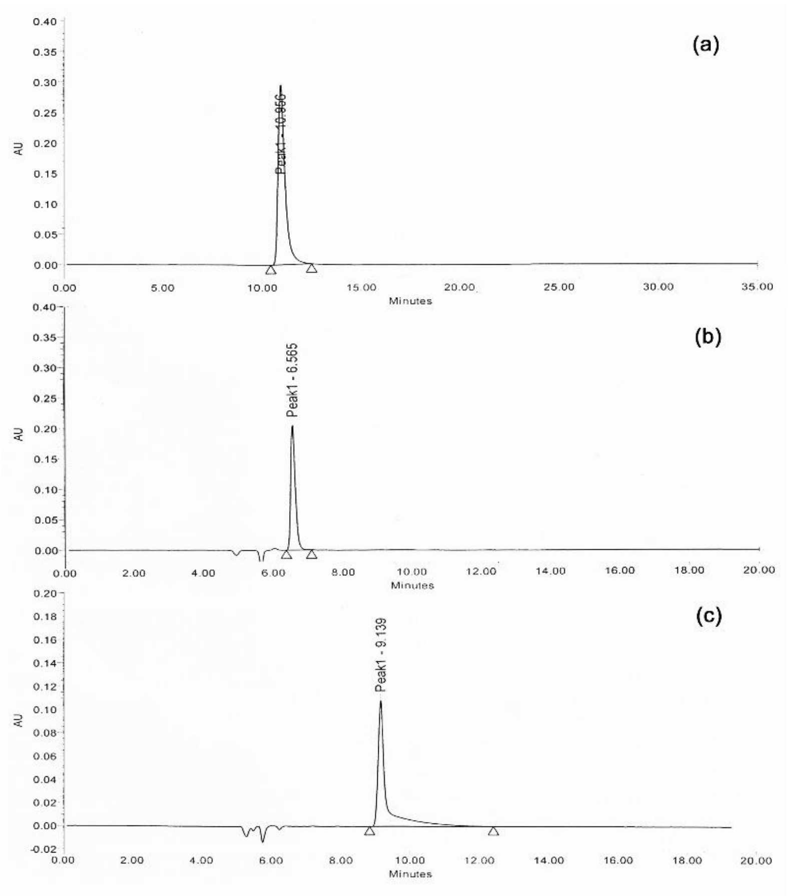

FIGURE S5**:** HPLC chromatograms of genipin using chiral columns under different conditions. (a) Entry 1; (b) Entry 2; (c) Entry 3.

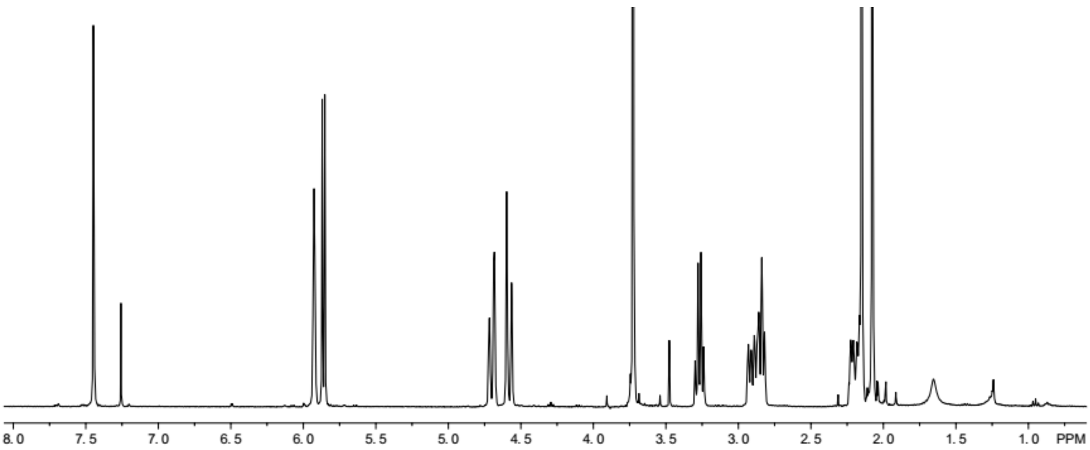

FIGURE S6**:** ^{1}H-NMR spectrum of acetylated genipin in C_5D_5N.

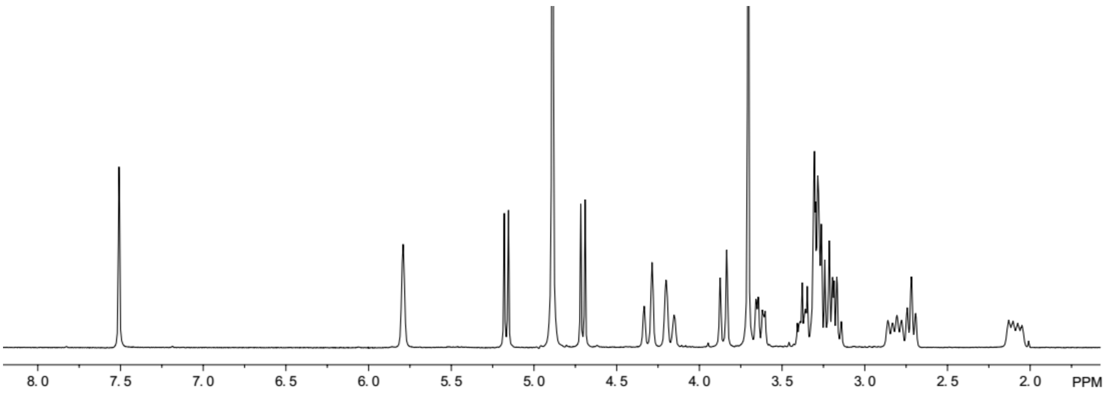

FIGURE S7**:** ^{1}H-NMR spectrum of geniposide in $CDCl_3$.

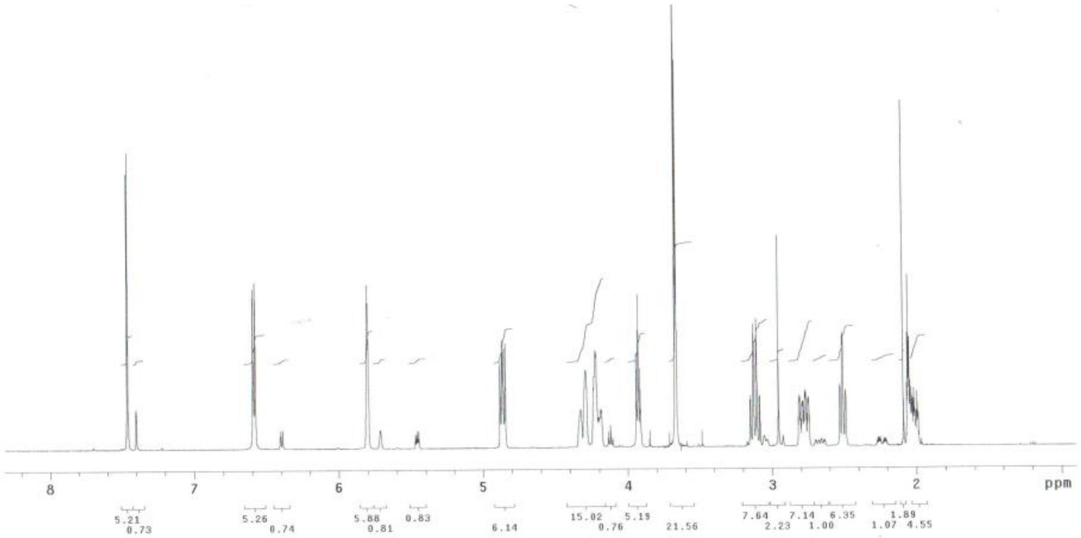

FIGURE S8**:** ^{1}H-NMR spectrum of genipin in DMCO-d_6.

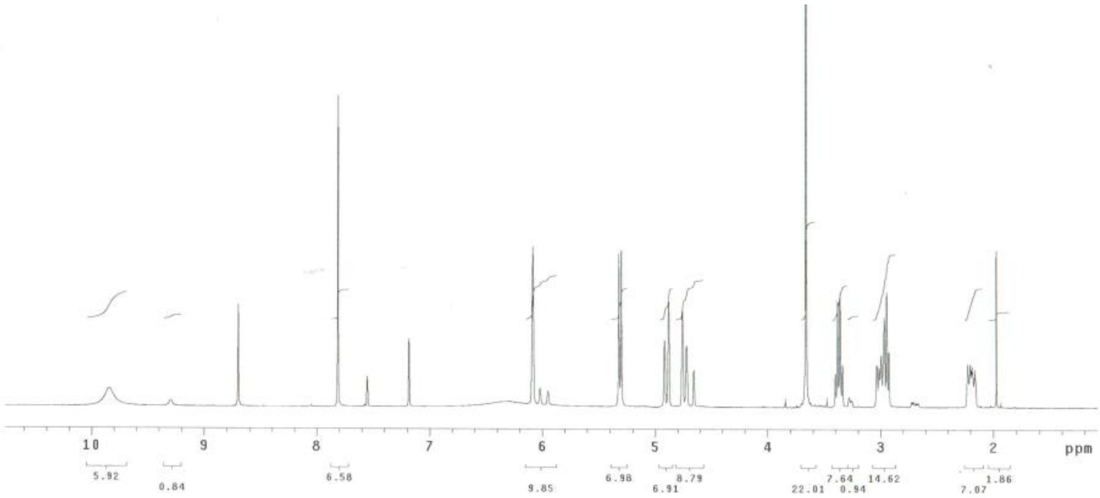

FIGURE S9**:** ^{1}H-NMR spectrum of genipin in C_5D_5N.

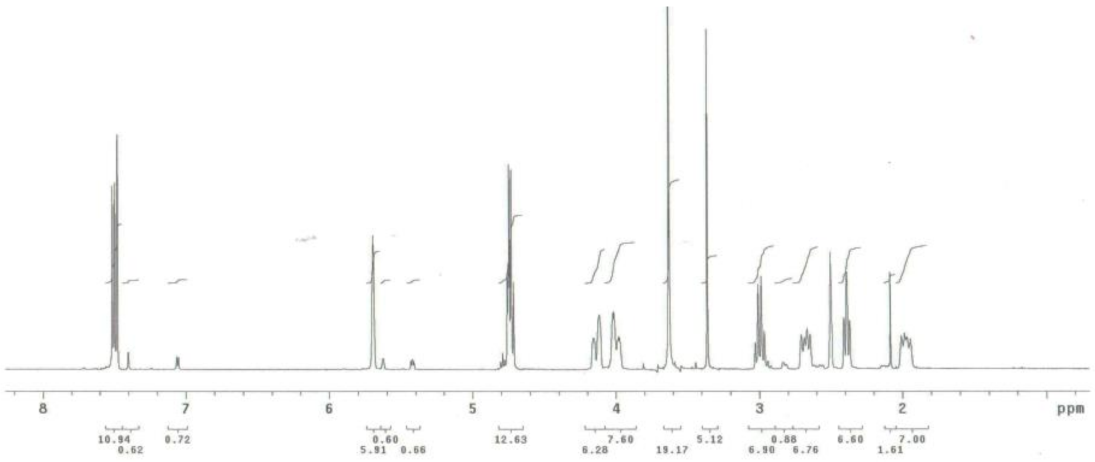

FIGURE S10**:** ^{1}H-NMR spectrum of genipin in DMSO-d_6.

TABLE S1: Crystallographic data of genipin.

Empirical formula	$C_{11}H_{14}O_5$
Formula weight	226.22
Temperature	293(2) K
Wavelength	0.71073 Å
Crystal system	monoclinic
Space group (number)	P2 (1)
Unit cell dimensions	a=7.563(7) Å
	b=17.799(17) Å
	c=8.151(8) Å
	α=90°
	β = 93.720(17)°
	γ = 90°
Volume	1094.9(19) $Å^3$
Z	4
Calculated density	1.372 mg/m^3
Absorption coefficient	0.109 mm^{-1}
F(000)	480
Crystal size	0.402 × 0.369 × 0.211 mm
θ range for data collection	2.29-25.50°
Limiting indices	–9 ≤–h ≤–9, –21 ≤–k ≤–20, –6 ≤–l≤–9
Reflections collected/ unique	5288/3614 [R(int)=0.0465]
Data/restrictions/parameters	3614/1/295
Refinement method	Full-matrix least-squares on F^2
Goodness-of-fit on F^2	1.083
Final R indices [I > 2σ(I)]	R1 = 0.0757; wR2 = 0.1930
R indices (all data)	R1 = 0.0882 wR2 = 0.2022
Largest diff. peak and hole	0.457 and –0.226 e·$Å^{-3}$
